# Retrospective Cohort Study of Effects of the COVID-19 Pandemic on Tuberculosis Notifications, Vietnam, 2020

**DOI:** 10.3201/eid2803.211919

**Published:** 2022-03

**Authors:** Tasnim Hasan, Viet Nhung Nguyen, Hoa Binh Nguyen, Thu Anh Nguyen, Hien T.T. Le, Cuong D. Pham, Nam Hoang, Phuong T.M. Nguyen, Justin Beardsley, Guy B. Marks, Greg J. Fox

**Affiliations:** The Woolcock Institute for Medical Research, Glebe, New South Wales, Australia (T. Hasan, T.A. Nguyen, H.T.T. Le, C.D. Pham, G.B. Marks, G.J. Fox);; University of Sydney, Sydney, New South Wales, Australia (T. Hasan, J. Beardsley, G.B. Marks, G.J. Fox);; National Lung Hospital, Hanoi, Vietnam (V.N. Nguyen, H.B. Nguyen, N.H. Do, P.T.M. Nguyen);; University of New South Wales, Sydney (G.B. Marks)

**Keywords:** COVID-19, respiratory infections, tuberculosis and other mycobacteria, severe acute respiratory syndrome coronavirus 2, SARS-CoV-2, SARS, coronavirus disease, zoonoses, viruses, coronavirus, Vietnam, bacteria

## Abstract

We evaluated the effects of the coronavirus disease pandemic on diagnosis of and treatment for tuberculosis (TB) in Vietnam. We obtained quarterly notifications for TB and multidrug-resistant/rifampin-resistant (MDR/RR) TB from 2015–2020 and evaluated changes in monthly TB case notifications. We used an interrupted time series to assess the change in notifications and treatment outcomes. Overall, TB case notifications were 8% lower in 2020 than in 2019; MDR/RR TB notifications were 1% lower. TB case notifications decreased by 364 (95% CI −1,236 to 508) notifications per quarter and MDR/RR TB by 1 (95% CI −129 to 132) notification per quarter. The proportion of successful TB treatment outcomes decreased by 0.1% per quarter (95% CI −1.1% to 0.8%) in 2020 compared with previous years. Our study suggests that Vietnam was able to maintain its TB response in 2020, despite the pandemic.

Since January 2020, severe acute respiratory syndrome coronavirus 2 (SARS-CoV-2) has been causing a global coronavirus disease (COVID-19) pandemic that has had wide-reaching effects on delivery of care for many other health conditions, including tuberculosis (TB). Each year, ≈10 million TB cases are diagnosed and ≈1.5 million TB deaths occur worldwide (*1*). The World Health Organization (WHO) has identified substantial effects of the COVID-19 pandemic on TB control efforts (*1*). By late 2020, substantial reductions in TB case notifications were evident in both high- and middle-income countries (*2*–*6*), including countries where COVID-19 had been well-controlled (*7*). Decreased TB notifications led to fears that delays in case detection and reduced treatment completion resulting from the COVID-19 pandemic might lead to increased *Mycobacterium tuberculosis* transmission and consequently higher mortality rates (*8*). Indeed, evidence suggests that the COVID-19 pandemic has resulted in reduced patient adherence to treatment (*9*), decreased access to medications (*10*,*11*), delayed access to services (*10*,*12*), and higher rates of loss to follow-up for patients with TB (*10*). Some of this disruption has been attributed to diversion of resources and interruptions to drug supply and delivery resulting from the COVID-19 pandemic (*13*). Furthermore, some persons with TB have avoided seeking healthcare because of fear of acquiring COVID-19 (*14*). In addition, evidence from South Africa suggests that outcomes for SARS-CoV-2 infection are worse for patients co-infected with TB (*15*).

Vietnam is a high-burden TB country and ranks among the top 30 high-burden countries for multidrug-resistant/rifampin-resistant (MDR/RR) TB (*16*). However, by the end of 2020, Vietnam had one of the lowest rates of reported COVID-19 cases in the region. Vietnam had its first confirmed COVID-19 case in January 2020; because of effective public health strategies, by the end of the year Vietnam had reported only ≈1,500 COVID-19 cases and 35 deaths (*17*,*18*). Early in 2020, localized outbreaks of COVID-19 occurred in Vietnam’s 2 largest cities, Hanoi and Ho Chi Minh City; subsequent outbreaks occurred in central Vietnam. The effect of Vietnam’s robust public health response against COVID-19 on TB case notifications is unknown. We aimed to evaluate the effect of the COVID-19 pandemic on TB case notifications and treatment outcomes during the first year of the pandemic in Vietnam by comparing programmatic data from 2020 to data for the preceding 5 years.

## Methods

### Study Design and Setting

We conducted a retrospective cohort study to compare national case notification and treatment outcomes for patients with TB and MDR/RR TB in Vietnam in 2020, the first year of the COVID-19 pandemic, with those from the preceding 5 years (2015–2019). Vietnam, located in Southeast Asia, has a population of 96 million and reports ≈100,000 TB cases and >11,000 TB deaths every year (*19*). Screening and treatment for TB are delivered by the National Tuberculosis Program (NTP) across all of Vietnam’s 63 provinces. Standardized TB treatment is delivered free of charge through district TB units and continuous treatment generally is supervised at home by family members. Patients routinely collect medication from health facilities at intervals between once a week and once a month. Changes to the delivery of care for TB patients during periods of physical distancing for COVID-19 included longer intervals between medication dispensing and increased intervals between microbiological testing and clinical review.

Two primary COVID-19 outbreaks occurred in Vietnam during 2020. The first outbreak occurred in April, with epicenters in Hanoi and Ho Chi Minh City. The second outbreak occurred during July–September 2020 in central Vietnam, primarily in Da Nang and Quang Nam provinces. In response to the pandemic, the government of Vietnam implemented strict public health policies, including mandatory quarantine for travelers and those with confirmed COVID-19 cases; facility-based isolation and testing of first-generation case-contacts and self-isolation for second-generation case-contacts; closing of schools and business; physical distancing policies; and public health messaging (*17*). Between COVID-19 surges, the NTP provided mobile community screening clinics to improve case detection and access to TB services for patients. These policies were enforced nationally in April 2020, and more localized policies targeting provinces with increased COVID-19 case numbers were implemented during July–September 2020. Between outbreaks, Vietnam had long periods in which no COVID-19 cases were reported, at times going several months reporting zero SARS-CoV-2 community transmission (*20*). Furthermore, 17 provinces reported no COVID-19 cases in 2020.

### Patient Eligibility and Data

#### TB Patients

**Figure 1:**
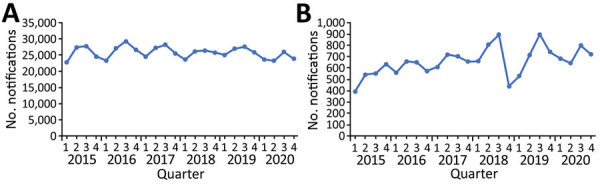
Quarterly tuberculosis notifications, Vietnam, 2015–2020. A) All tuberculosis notifications. B) Multidrug-resistant/rifampin-resistant tuberculosis notifications.

We included patients of all ages who began TB treatment through the NTP during January 2015–December 2020. All persons with confirmed TB were recorded by district and by date of enrollment into TB treatment. Reported data include age, sex, prior treatment history, diagnostic test results, antimicrobial drug regimen, and treatment outcomes reported according to WHO standard definitions (*21*). We evaluated the number of quarterly TB notifications during 2015–2020 (Figure 1, panel A). WHO-defined treatment outcomes were reported for cases during 2016–2020. Cases notified outside the NTP, for example through private sector healthcare, comprised only a small portion (<10%) of all TB and MDR/RR TB cases and we did not include these cases in this study. However, cases reported outside NTP account for discrepancies between total notifications in this study compared with WHO reports.

#### MDR/RR TB Patients

We identified patients who began treatment for MDR/RR TB (defined as TB with resistance to isoniazid and rifampin) through a separate national database. We included MDR/RR TB case notifications during January 2015–December 2020 (Figure 1, panel B). Reported data include underlying conditions, site of TB disease, smear and culture status at diagnosis, drug resistance, and adverse events. Patients with diagnosed MDR/RR TB underwent treatment according to WHO guidelines, comprising either 9-month or 20-month standardized antimicrobial drug regimens (*22*). NTP reported quarterly MDR/RR TB notifications during 2015–2020. 

### Data Analysis

**Figure 2:**
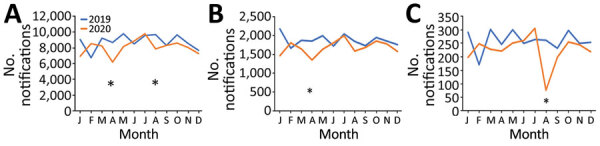
Change in number of monthly tuberculosis notifications during the COVID-19 pandemic, Vietnam, 2019–2020. A) Vietnam; B) Hanoi and Ho Chi Minh City; C) Da Nang and Quang Nam Provinces. Asterisks indicate timing of COVID-19 outbreaks. COVID-19, coronavirus disease.

Patient-level data were only available for 2019 and 2020. We summarized TB notifications by age, sex, history of previous treatment, and treatment outcome and reported proportions of missing data (Table 1), as well as monthly TB notifications for 2019 and 2020 (Figure 2), including the percentage change in monthly and yearly notifications (Appendix Table). We also calculated the monthly notifications and percentage change in notifications from cities and provinces where COVID-19 outbreaks occurred, Ho Chi Minh City and Hanoi in April 2020 and Da Nang and Quang Nam in July–August 2020 (Figure 2; Appendix Table 1). For comparison, we chose 2 provinces in South and Central Vietnam where no COVID-19 cases were detected during the study period, Can Tho and Nghe An (Appendix Figure).

**Table 1:** Characteristics of persons with diagnosed tuberculosis, Vietnam, 2019 and 2020*

Characteristics	2019	2020
Total no. cases notified	105,680	96,998
Age group, y		
<20	5,371 (5.1)	4,378 (4.5)
20–39	32,962 (31.2)	29,303 (30.2)
40–59	39,177 (37.1)	36,094 (37.2)
60–79	23,942 (22.7)	23,121 (23.8)
>80	4,228 (4.0)	4,102 (4.2)
Sex†		
M	74,331 (70.3)	68,737 (70.9)
F	30,248 (28.6)	27,482 (28.3)
Region‡		
North	26,352 (24.9)	23,862 (24.6)
Central	18,969 (18.0)	16,329 (16.8)
South	59,256 (56.1)	56,027 (57.8)
Registration group§		
New diagnosis	96,445 (91.3)	89,048 (91.8)
Relapse	6,575 (6.2)	5,895 (6.1)
Retreatment	1941 (1.8)	1,812 (1.9)

*Values represent no. (%) except as indicated. Definitions for classification according to World Health Organization guidelines (https://www.who.int/tb/publications/definitions/en). †Sex was not reported for 1,101 (1.0%) cases in 2019 and 779 (0.8%) cases in 2020. ‡District and region were not reported for 1,103 (1.0%) cases in 2019 and 780 (0.8%) cases in 2020. §Registration group was not reported for 719 (0.7%) cases in 2019 and 243 (0.3%) cases in 2020.

For quarterly TB notifications during 2015–2020, we used an interrupted time series (*23*) to determine whether quarterly TB notifications decreased during January–June 2020, compared with quarterly notifications during 2015–2019. We used an interrupted time series because it enables a comparison of the change in the trend of an event before and after an interruption, in this case COVD-19. We also used an interrupted time series to determine whether the proportion of patients with treatment success changed for patients beginning treatment during July 2019–January 2020 compared with patients commencing treatment during January 2016–June 2019 (Table 2). Patients beginning first-line treatment for TB during July–December 2019 were scheduled to finish treatment during January–June 2020, after the onset of the COVID-19 pandemic.

**Table 2:** Tuberculosis treatment outcomes for patients receiving first-line therapy, stratified by date treatment began, Vietnam*

Treatment start date	Favorable outcome†	Failure	Lost to follow-up	Death	Transfer to MDR‡	Not evaluated	Total unfavorable outcome§
2016							
Q1	18,405 (92.9)	146 (0.7)	434 (2.2)	545 (2.8)	31 (0.2)	252 (1.3)	1,156 (5.8)
Q2	20,943 (93.3)	129 (0.6)	567 (2.5)	531 (2.4)	47 (0.2)	235 (1.0)	1,274 (5.7)
Q3	22,656 (93.5)	139 (0.6)	580 (2.4)	535 (2.2)	56 (0.2)	258 (1.1)	1,310 (5.4)
Q4	24,106 (91.6)	192 (0.7)	700 (2.7)	667 (2.5)	103 (0.4)	562 (2.1)	1,662 (6.3)
Total 2016	86,110 (92.8)	606 (0.7)	2,281 (2.5)	2,278 (2.5)	237 (0.3)	1,307 (1.4)	5,402 (5.8)
2017							
Q1	22,453 (91.0)	193 (0.8)	676 (2.7)	557 (2.3)	103 (0.4)	694 (2.8)	1,529 (6.2)
Q2	24,863 (91.5)	188 (0.7)	686 (2.5)	632 (2.3)	102 (0.4)	715 (2.6)	1,608 (5.9)
Q3	25,813 (92.3)	170 (0.6)	712 (2.5)	615 (2.2)	107 (0.4)	549 (2.0)	1,604 (5.7)
Q4	23,171 (91.6)	119 (0.5)	652 (2.6)	613 (2.4)	120 (0.5)	615 (2.4)	1,504 (5.9)
Total 2017	96,300 (91.6)	670 (0.6)	2,726 (2.6)	2,417 (2.3)	432 (0.4)	2,573 (2.4)	6,245 (5.9)
2018							
Q1	21,514 (90.9)	164 (0.7)	624 (2.6)	669 (2.8)	114 (0.5)	573 (2.4)	1,571 (6.6)
Q2	23,942 (91.4)	129 (0.5)	691 (2.6)	648 (2.5)	159 (0.6)	623 (2.4)	1,627 (6.2)
Q3	24,221 (91.6)	135 (0.5)	668 (2.5)	640 (2.4)	127 (0.5)	657 (2.5)	1,570 (5.9)
Q4	23,575 (91.1)	122 (0.5)	691 (2.7)	615 (2.4)	108 (0.4)	758 (2.9)	1,536 (5.9)
Total 2018	93,252 (91.3)	550 (0.5)	2,674 (2.6)	2,572 (2.5)	508 (0.5)	2,611 (2.6)	6,304 (6.2)
2019							
Q1	21,842 (90.4)	144 (0.6)	748 (3.1)	624 (2.6)	110 (0.5)	701 (2.9)	1,626 (6.7)
Q2	24,122 (90.7)	155 (0.6)	777 (2.9)	701 (2.6)	150 (0.6)	680 (2.6)	1,783 (6.7)
Q3	25,525 (91.3)	123 (0.4)	784 (2.8)	632 (2.3)	183 (0.7)	724 (2.6)	1,722 (6.2)
Q4	23,501 (91.0)	124 (0.5)	681 (2.6)	614 (2.4)	166 (0.6)	729 (2.8)	1,585 (6.1)
Total 2019	73,148 (91.0)	402 (0.5)	2,242 (2.8)	1,947 (2.4)	499 (0.6)	2,133 (2.7)	5,090 (6.3)
2020¶							
Q1	21,613 (91.2)	144 (0.6)	516 (2.2)	643 (2.7)	156 (0.7)	623 (2.6)	1,459 (6.2)

*Values are no. (%). MDR, multidrug-resistant; Q, quarter. †Favorable outcomes include cure and treatment complete. ‡Cases were transferred to MDR status when resistance testing revealed MDR TB or treatment with first-line antimicrobial drugs failed. §Unfavorable outcomes include failure, loss to follow-up, death, and transfer to MDR. ¶For 2020, only outcomes for Q1 were available.

For 2019 and 2020 data, we summarized MDR/RR TB notifications by age, sex, history of previous treatment, smear and culture results, and treatment outcome (Table 3). We noted proportions of missing data. We summarized monthly MDR/RR TB notifications made during 2019 and 2020, including the percentage change in monthly and yearly notifications, and separately calculated the difference in notifications for Ho Chi Minh City and Hanoi (Appendix Table 2). We used an interrupted time series to determine whether quarterly MDR/RR TB notifications decreased during 2020 compared with 2015–2019.

**Table 3:** Characteristics of patients diagnosed with MDR/RR TB by the Vietnam National Tuberculosis Program, 2019 and 2020*

Characteristics	No. (%) cases notified	
	2019	2020
Total MDR/RR TB cases	2,889	2,851
Age group, y		
<20	124 (4.3)	71 (2.5)
20–39	1,083 (37.5)	1,072 (37.6)
40–59	1,237 (42.8)	1,221 (42.8)
60–79	414 (14.3)	455 (16.0)
≥80	31 (1.1)	32 (1.1)
Sex		
M	2,206 (76.4)	2,185 (76.6)
F	683 (23.6)	666 (23.4)
Registration group†		
New	1,059 (36.7)	1,258 (44.1)
Relapse	1,012 (35.0)	976 (34.2)
Failure	328 (11.4)	210 (7.4)
Transfer in	4 (0.1)	2 (0.0)
Transfer after default	154 (5.3)	125 (4.4)
Other	227 (7.9)	177 (6.2)
No. previous treatment episodes		
1	1,011 (35.0)	855 (30.0)
2	199 (6.9)	169 (5.9)
3	34 (1.1)	34 (1.2)
4	4 (0.1)	6 (0.2)
5	1 (0.0)	2 (0.0)
Smear status at diagnosis‡		
Negative	568 (19.7)	532 (18.7)
Scanty	289 (10.0)	244 (8.6)
1+	415 (14.4)	428 (15.0)
2+	308 (10.7)	261 (9.2)
3+	275 (9.5)	262 (9.2)
Culture status at diagnosis§		
Negative	132 (4.6)	91 (3.2)
Positive	675 (23.4)	564 (19.8)
Contaminated	14 (0.5)	12 (0.4)
Underlying conditions		
HIV	119 (4.1)	82 (2.9)
Diabetes	47 (1.6)	54 (1.9)
COPD	7 (0.2)	7 (0.2)
Chronic kidney disease	11 (0.4)	7 (0.2)
Cardiac disease	11 (0.4)	24 (0.8)
Baseline antimicrobial drug resistance*¶*		
Monoresistance#	301 (10.4)	260 (9.1)
Polydrug resistance	122 (4.2)	94 (3.3)
MDR TB	1,545 (53.5)	1,666 (58.4)
Pre-XDR TB	47 (1.6)	47 (1.6)
XDR TB	12 (0.4)	10 (0.4)
Site of disease**		
Extrapulmonary	119 (4.1)	131 (4.6)
Pulmonary††	2,619 (90.7)	2,592 (90.9)

*COPD, chronic obstructive pulmonary disease; MDR, multidrug-resistant; MDR/RR, multidrug-resistant/rifampin-resistant; TB, tuberculosis; XDR, extensively drug resistant. †Registration group was not reported for 105 (3.6%) cases in 2019 and 103 (3.6%) cases in 2020. ‡Smear status was not reported for 1,034 (35.8%) cases in 2019 and 1,124(39.4%) cases in 2020. §Culture status was not reported for 2,068 (71.6%) cases in 2019 and 2,184 (76.6%) cases in 2020. ¶Baseline resistance was not reported for 862 (29.8%) cases in 2019 and 774 (27.1%) cases in 2020. #Site of disease was not reported for 151 (5.2%) cases in 2019 and 128 (4.5%) cases in 2020. **Definitions for classification according to World Health Organization guidelines (https://www.who.int/tb/publications/definitions/en). ††Pulmonary TB includes patients with pulmonary TB alone and patients with pulmonary and extra-pulmonary TB.

For MDR/RR TB, we calculated the relative risk for cases to have a positive smear diagnosis in 2020 compared with 2019. Similarly, we calculated the relative risk for a positive culture diagnosis in 2020 compared with 2019.

We calculated CIs and performed analyses by using SAS version 9.4 (SAS Institute, Inc., https://www.sas.com). The University of Sydney provided ethics approval for this study (approval no. HREC 2020/353). The study also was approved by the Vietnam National Lung Hospital.

## Results

### TB Notifications

NTP reported 105,680 TB cases in 2019 and 96,998 in 2020 (Table 1). Most cases were diagnosed among male persons, and most cases were notified in the south of the country.

Overall, national TB case notifications dropped by 8% during 2020 compared with 2019 (Appendix Table 1). In April 2020, during the first COVID-19 outbreak in Vietnam, we observed a 29% decrease in national TB notifications compared with April 2019 (Appendix Table 1). We also noted a decrease in case notifications during January 2020 compared with January 2019. This difference likely reflects the difference in the date of the Lunar New Year, which was earlier in 2020 than 2019, rather than an effect of the pandemic. In Hanoi and Ho Chi Minh City, areas most affected during this outbreak, the difference in TB notifications was 27% (Appendix Table 1). During the second major COVID-19 outbreak in August 2020, TB notifications declined by 19% nationally and 71% in the provinces most affected, Da Nang and Quang Nam, compared with August 2019 (Appendix Table 1). Although a pronounced decrease in TB notifications was not observed in the 2 provinces with no COVID-19 cases, Can Tho and Nghe An, we did note a 7%–16% decrease in annual TB notifications in these provinces for 2020 compared with 2019 (Appendix Table 1).

TB notifications decreased by 364 notifications per quarter (95% CI −1,236 to 508) during the year after the onset of the COVID-19 compared with the previous 5 years. Successful TB treatment outcomes decreased by 0.1% per quarter (95% CI −1.1% to 0.8%) for patients completing treatment in 2020, compared with rates for 2016–2019 (Appendix Table 3).

### MDR/RR TB Notifications

We noted all known MDR/RR TB cases reported in Vietnam during 2015–2020 (Appendix Table 4). In 2019, 2,889 MDR/RR TB cases were notified to the electronic TB manager; 2,851 cases were notified in 2020. We noted patient demographics, treatment history, and treatment outcomes between the 2 years (Table 3). 

**Figure 3:**
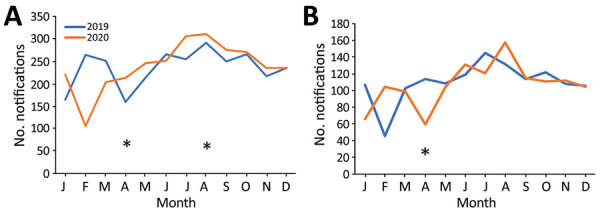
Change in number of monthly notifications for multidrug-resistant/rifampin-resistant tuberculosis during the COVID-19 pandemic, Vietnam, 2019–2020. A) Vietnam; B) Hanoi and Ho Chi Minh City. Asterisks indicate timing of COVID-19 outbreaks. COVID-19, coronavirus disease.

In April 2020, during the first major COVID-19 outbreak and the first nationally implemented social distancing efforts, MDR/RR TB notifications decreased by 27% compared with notifications during April 2019 (Figure 3; Appendix Table 3). Hanoi and Ho Chi Minh City, which were most affected during this outbreak, contributed >40% of national TB case notifications, but the combined number of notified MDR/RR TB cases in these 2 cities decreased by 47% (Appendix Table 3). However, overall MDR/RR TB notifications decreased by just 1% in 2020 compared with 2019. We observed no difference in the proportion of notified patients with smear-positive TB compared with smear-negative TB (risk ratio 1.00, 95% CI 0.96–1.05), or culture-positive TB compared with culture-negative TB (risk ratio 1.03, 95% CI 0.99–1.08) between 2020 and 2019 (Table 3). The difference in MDR/RR TB notifications decreased by 1 notification per quarter (95% CI −129 to 132) after the start of the COVID-19 pandemic compared with before the pandemic (Appendix Table 2). 

## Discussion

This retrospective cohort study compared the number of notified TB cases and treatment outcomes in Vietnam during the first year of the COVID-19 pandemic with those during the preceding 5 years. We found an 8% decrease in overall TB notifications and a 1% decrease in MDR/RR TB notifications in 2020 compared with the preceding year. We did not observe any difference in TB treatment outcomes in 2020 compared with the period 2016–2019. However, we did see noticeable decreases in TB and MDR/RR TB case notifications in the provinces affected most by COVID-19 in the months in which social distancing measures were enforced. This observation suggests a possible delay in the diagnosis of TB and MDR/RR TB cases. NTP and provincial TB programs in areas most affected by COVID-19 should develop strategies to reduce the delay in diagnosis and prevent community transmission.

Our study starkly contrasts findings from other settings during the COVID-19 pandemic. Several countries reported ≤30% fewer TB notifications during the first half of 2020 than before COVID-19 (*2*,*4*,*7*,*9*,*24*,*25*). In Malawi, one province noted a 36% decrease in notifications, after which a subsequent rebound in notifications occurred by the end of the year, culminating in a 24% overall decrease in TB notifications in 2020 (*26*). Similarly, the United States reported an overall 20% decrease in TB notifications for 2020 compared with those for the previous year (*4*), although some of the reduction in low-prevalence settings might be due to reduced immigration from high-prevalence settings (*4*). The 8% decrease in TB notifications we observed in Vietnam was modest compared to these other settings. Effective control of the COVID-19 pandemic likely enabled health services to operate and compensate during non-lockdown periods. Although we found a decrease in TB notifications for most months in 2020 compared with those for 2019, the decrease in TB notifications was modest during months without surges in COVID-19 case numbers, including June, November, and December (Figure 2).

In 2020, during the first COVID-19 outbreak, Vietnam implemented nationwide physical distancing and public health policies. These restrictions lasted <2 months, after which daily life returned to normal for most of the population (*27*). Our study confirms that the largest decrease in case notifications for both TB and MDR/RR TB nationally was noted during this period. However, case notifications rebounded in subsequent months, resulting in the limited reduction observed in overall case notifications for the year. Nevertheless, substantial transient downturns in case notification were observed during these short periods of physical restrictions in hotspot areas, and we noted a 70% decrease in TB notifications in the 2 provinces most affected by the second outbreak. The findings overall confirm that TB notifications were adversely affected during COVID-19 outbreaks and periods in which lockdown was enforced to control the pandemic, even in the absence of COVID-19 cases. Factors contributing to the reduced TB notifications likely include difficulty accessing healthcare (*10*,*12*) and fear of catching COVID-19 at healthcare facilities (*14*).

Our findings mirror findings in neighboring China, where the incidence of COVID-19 remained low amidst a moderately high incidence of TB. Data from the first half of 2020 in China showed that TB notifications also rebounded in the months after the easing of initial COVID-19 restrictions (*28*). Our findings and those from China suggest that when COVID-19 outbreaks are relatively brief, losses in TB notifications can be compensated for in subsequent months. However, increased TB surveillance is required after periods of strict lockdown to identify transmission that can occur during delayed case finding. Delays in TB case finding also are suggested by observational studies that demonstrated fewer sputum samples submitted for TB smear and culture during 2020 than 2019 (*29*,*30*).

Furthermore, delayed case finding could result in more advanced TB disease before diagnosis. We found no difference in the proportion of patients being seen with more advanced TB disease in health facilities, measured by culture and smear status, after a period of social restrictions. Another study in South Korea also found no difference in smear status, culture results, or treatment adherence for TB patients between the first 6 months of 2020 and the year preceding the pandemic (*31*). Both Vietnam and South Korea had smaller COVID-19 outbreaks, measured as total cases and per capita, in 2020 compared with other settings globally (*18*). However, a much smaller study in Spain, a country with a high COVID-19 burden, reported more advanced radiologic findings for TB notifications in 2020 (*32*). Further global data from settings with high COVID-19 burdens will be needed to appreciate the effects of the COVID-19 pandemic on delayed TB case finding.

We found no difference in treatment outcomes for TB patients who started treatment in the 6 months before the pandemic (July–December 2019) and completed treatment during the pandemic compared with TB patients beginning treatment during 2016–July 2019. In contrast, Italy, a country with low TB incidence, reported a substantial increase in the proportion of patients experiencing poor TB treatment outcomes during the pandemic, including loss to follow-up and death (*33*).

A strength of our study is that we used a comprehensive national database that can be generalizable at a national level for Vietnam. We evaluated TB notifications during the COVID-19 pandemic compared with TB notification data over a prolonged period (2016–2019) before the pandemic, which enabled us to account for trends over time; single comparisons might miss previously existing trends, including seasonal variation (*34*).

Our study is limited by using routinely collected programmatic data, which is limited to key information about patients and only includes the 2020 calendar year. Collection of smear status was missing for ≈30% of cases, and culture status at baseline was missing in ≈70%, limiting the ability to fully appreciate any change in smear or culture status between 2019 and 2020. Furthermore, because the duration of MDR/RR TB treatment is 9 or 20 months, we could only compare treatment outcomes for patients on standard first-line therapy. Finally, the effects on treatment outcomes can only be fully appreciated when all patients who commenced treatment in 2019 and 2020 receive an outcome.

Several policy implications arise from this study. Evidence suggests that countries with prolonged control of community transmission of SARS-CoV-2, such as China, Vietnam, and South Korea, have seen only modest impacts on overall TB notifications. Furthermore, evidence also suggests that TB notifications can rebound after COVID-19 has been controlled. Thus, involvement of national and international organizations in the care of TB patients is critical for monitoring and evaluating the interactions between COVID-19 and health priorities, preparing the healthcare sector, and limiting service disruptions. The COVID-19 pandemic is far from over and must be controlled before care to other infectious diseases such as TB can be restored.

Future studies could address the effect of a prolonged COVID-19 pandemic on delayed TB diagnosis, especially in settings with a high burden of COVID-19. The COVID-19 pandemic has taken a markedly different course from mid-2021, and Vietnam has experienced major outbreaks nationwide because of the Delta variant. Further research evaluating this period will enable us to contrast the effects of COVID-19 outbreaks on TB notifications over the course of the pandemic. Further evaluation also is needed to assess effects of COVID-19 on TB treatment outcomes, including changes in adverse TB outcomes, such as loss to follow-up due to decreased access to healthcare systems. Ultimately, the extent of the effects of the COVID-19 pandemic on TB care will take many years to fully appreciate, both in Vietnam and globally. Operational research is required to continue to identify these effects and to maintain resources for TB programs despite competing healthcare priorities. Finally, COVID-19–related restrictions, such as social distancing and the use of facemasks, might limit TB transmission; however, the adverse consequences of the COVID-19 pandemic likely are not adequately offset by these beneficial effects, and this requires further exploration.

In conclusion, our study demonstrated a very limited decrease in TB notifications in Vietnam during the first year of the COVID-19 pandemic, despite national physical distancing measures. Settings with high rates of community transmission of SARS-CoV-2 are likely to experience a surge in TB notifications when COVID-19 restrictions are eased. These settings should increase healthcare capacity to detect and treat TB cases missed during COVID-19 restrictions.

Appendix Additional information on the effects of the COVID-19 pandemic on tuberculosis notifications, Vietnam, 2020.

## References

[1] World Health Organization. Tuberculosis [cited 2021 Apr 5]. https://www.who.int/news-room/fact-sheets/detail/tuberculosis

[2] Buonsenso D, Iodice F, Sorba Biala J, Goletti D. COVID-19 effects on tuberculosis care in Sierra Leone. Pulmonology. 2021;27:67–9. 10.1016/j.pulmoe.2020.05.013 32561353 PMC7275172

[3] Daw MA, Zgheel FA, El-Bouzedi A, Ahmed MO. Spatiotemporal distribution of tuberculosis and COVID-19 during the COVID-19 pandemic in Libya. Disaster Med Public Health Prep. 2021;15:e43–5. 10.1017/dmp.2020.458 33208222 PMC7884655

[4] Deutsch-Feldman M, Pratt RH, Price SF, Tsang CA, Self JL. Tuberculosis - United States, 2020. MMWR Morb Mortal Wkly Rep. 2021;70:409–14. 10.15585/mmwr.mm7012a1 33764959 PMC7993554

[5] Louie JK, Reid M, Stella J, Agraz-Lara R, Graves S, Chen L, et al. A decrease in tuberculosis evaluations and diagnoses during the COVID-19 pandemic. Int J Tuberc Lung Dis. 2020;24:860–2. 10.5588/ijtld.20.0364 32912395

[6] Odume B, Falokun V, Chukwuogo O, Ogbudebe C, Useni S, Nwokoye N, et al. Impact of COVID-19 on TB active case finding in Nigeria. Public Health Action. 2020;10:157–62. 10.5588/pha.20.0037 33437681 PMC7790486

[7] Lai C-C, Yu W-L. The COVID-19 pandemic and tuberculosis in Taiwan. J Infect. 2020;81:e159–61. 10.1016/j.jinf.2020.06.014 32534000 PMC7286835

[8] Cilloni L, Fu H, Vesga JF, Dowdy D, Pretorius C, Ahmedov S, et al. The potential impact of the COVID-19 pandemic on the tuberculosis epidemic a modelling analysis. EClinicalMedicine. 2020;28:100603. 10.1016/j.eclinm.2020.100603 33134905 PMC7584493

[9] Ferrer JP, Suzuki S, Alvarez C, Berido C, Caballero M, Caraig B, et al. Experiences, challenges and looking to the future in a clinical tuberculosis cohort in the time of COVID-19 in the Philippines. Trans R Soc Trop Med Hyg. 2021;115:579–82. 10.1093/trstmh/trab025 33693916 PMC7989158

[10] Rai DK, Kumar R, Pandey SK. Problems faced by tuberculosis patients during COVID-19 pandemic: Urgent need to intervene. Indian J Tuberc. 2020;67(4S):S173–4. 10.1016/j.ijtb.2020.07.013 33308666 PMC7372252

[11] Udwadia ZF, Sharma S, Mullerpattan JB, Gajjar I, Pinto L. Effective use of telemedicine in Mumbai with a cohort of extensively drug-resistant “XDR” tuberculosis patients on bedaquiline during COVID-19 pandemic. Lung India. 2021;38:98–9. 10.4103/lungindia.lungindia_464_20 33402651 PMC8066929

[12] Cronin AM, Railey S, Fortune D, Wegener DH, Davis JB. Notes from the field: effects of the COVID-19 response on tuberculosis prevention and control efforts—United States, March–April 2020. MMWR Morb Mortal Wkly Rep. 2020;69:971–2. 10.15585/mmwr.mm6929a4 32701944 PMC7377818

[13] Jain VK, Iyengar KP, Samy DA, Vaishya R. Tuberculosis in the era of COVID-19 in India. Diabetes Metab Syndr. 2020;14:1439–43. 10.1016/j.dsx.2020.07.034 32755848 PMC7387287

[14] Ahmed SAKS, Ajisola M, Azeem K, Bakibinga P, Chen Y-F, Choudhury NN, et al.; Improving Health in Slums Collaborative. Impact of the societal response to COVID-19 on access to healthcare for non-COVID-19 health issues in slum communities of Bangladesh, Kenya, Nigeria and Pakistan: results of pre-COVID and COVID-19 lockdown stakeholder engagements. BMJ Glob Health. 2020;5:e003042. 10.1136/bmjgh-2020-003042 32819917 PMC7443197

[15] Boulle A, Davies M-A, Hussey H, Ismail M, Morden E, Vundle Z, et al. Risk factors for COVID-19 death in a population cohort study from the Western Cape Province, South Africa. Clin Infect Dis. 2021;73:e2005–15 . 32860699 10.1093/cid/ciaa1198 PMC7499501

[16] World Health Organization. Use of high burden country lists for TB by WHO in the post-2015 era: summary [cited 2021 Apr 5]. https://www.who.int/tb/publications/global_report/high_tb_burdencountrylists2016-2020summary.pdf

[17] Thanh HN, Van TN, Thu HNT, Van BN, Thanh BD, Thu HPT, et al. Outbreak investigation for COVID-19 in northern Vietnam. Lancet Infect Dis. 2020;20:535–6. 10.1016/S1473-3099(20)30159-6 32145188 PMC7158986

[18] John Hopkins University. COVID-19 dashboard by the Center for Systems Science and Engineering (CSSE) at Johns Hopkins University [cited 2021 Mar 1]. https://coronavirus.jhu.edu/map.html

[19] Nhung NV, Hoa NB, Khanh PH, Hennig C. Tuberculosis case notification data in Viet Nam, 2007 to 2012. Western Pac Surveill Response J. 2015;6:7–14. 10.5365/wpsar.2014.5.2.005 25960918 PMC4410103

[20] Nguyen TA, Nguyen BTC, Duong DT, Marks GB, Fox GJ. Experience in responding to COVID-19 outbreaks from Vietnam. Lancet Reg Health West Pac. 2021;7:100077. 10.1016/j.lanwpc.2020.100077 33532745 PMC7843250

[21] World Health Organization. Definitions and reporting framework for tuberculosis—2013 revision (updated December 2014 and January 2020). Geneva: The Organization; 2020.

[22] World Health Organization. WHO consolidated guidelines on drug-resistant tuberculosis treatment. Geneva: The Organization; 2019. 30946559

[23] Bernal JL, Cummins S, Gasparrini A. Interrupted time series regression for the evaluation of public health interventions: a tutorial. Int J Epidemiol. 2017;46:348–55. 27283160 10.1093/ije/dyw098 PMC5407170

[24] Shrinivasan R, Rane S, Pai M. India’s syndemic of tuberculosis and COVID-19. BMJ Glob Health. 2020;5:e003979. 10.1136/bmjgh-2020-003979 33199280 PMC7670552

[25] Golandaj JA. Insight into the COVID-19 led slow-down in TB notifications in India. Indian J Tuberc. 2021;68:142–5. 10.1016/j.ijtb.2020.12.005 33641836 PMC7745308

[26] Soko RN, Burke RM, Feasey HRA, Sibande W, Nliwasa M, Henrion MYR, et al. Effects of coronavirus disease pandemic on tuberculosis notifications, Malawi. Emerg Infect Dis. 2021;27:1831–9. 10.3201/eid2707.210557 34152962 PMC8237899

[27] Thai PQ, Rabaa MA, Luong DH, Tan DQ, Quang TD, Quach H-L, et al.; OUCRU COVID-19 Research Group. The first 100 days of severe acute respiratory syndrome coronavirus 2 (SARS-CoV-2) control in Vietnam. Clin Infect Dis. 2021;72:e334–42. 10.1093/cid/ciaa1130 32738143 PMC7454342

[28] Chen H, Zhang K. Insight into the impact of the COVID-19 epidemic on tuberculosis burden in China. Eur Respir J. 2020;56:2002710. 10.1183/13993003.02710-2020 32703778 PMC7397949

[29] Komiya K, Yamasue M, Takahashi O, Hiramatsu K, Kadota J-I, Kato S. The COVID-19 pandemic and the true incidence of Tuberculosis in Japan. J Infect. 2020;81:e24–5. 10.1016/j.jinf.2020.07.004 32650109 PMC7338857

[30] Nikolayevskyy V, Holicka Y, van Soolingen D, van der Werf MJ, Ködmön C, Surkova E, et al.; ERLTB-Net-2 study participants. Impact of the COVID-19 pandemic on tuberculosis laboratory services in Europe. Eur Respir J. 2021;57:2003890. 10.1183/13993003.03890-2020 33184119 PMC7670866

[31] Min J, Kim HW, Koo HK, Ko Y, Oh JY, Kim J, et al. Impact of COVID-19 pandemic on the national PPM tuberculosis control project in Korea: the Korean PPM monitoring database between July 2019 and June 2020. J Korean Med Sci. 2020;35:e388. 10.3346/jkms.2020.35.e388 33169559 PMC7653169

[32] Aznar ML, Espinosa-Pereiro J, Saborit N, Jové N, Sánchez Martinez F, Pérez-Recio S, et al. Impact of the COVID-19 pandemic on tuberculosis management in Spain. Int J Infect Dis. 2021;108:300–5. 10.1016/j.ijid.2021.04.075 33930543 PMC8078060

[33] Magro P, Formenti B, Marchese V, Gulletta M, Tomasoni LR, Caligaris S, et al. Impact of the SARS-CoV-2 epidemic on tuberculosis treatment outcome in Northern Italy. Eur Respir J. 2020;56:2002665. 10.1183/13993003.02665-2020 32703780 PMC7377210

[34] Bonell A, Contamin L, Thai PQ, Thuy HTT, van Doorn HR, White R, et al. Does sunlight drive seasonality of TB in Vietnam? A retrospective environmental ecological study of tuberculosis seasonality in Vietnam from 2010 to 2015. BMC Infect Dis. 2020;20:184. 10.1186/s12879-020-4908-0 32111195 PMC7048025

