# Supplementary material for: Retrospective Cohort Study of Effects of the COVID-19 Pandemic on Tuberculosis Notifications, Vietnam, 2020
Source: Emerg Infect Dis. 2022 Mar;28(3):684–92. doi: 10.3201/eid2803.211919 (PMC8888245; doi:10.3201/eid2803.211919)
Supplement: Appendix — Additional information on the effects of the COVID-19 pandemic on tuberculosis notifications, Vietnam, 2020. [file 21-1919-Techapp-s1.pdf]

Article DOI: https://doi.org/10.3201/eid2803.211919

Retrospective Cohort Study of Effects of the COVID-19 Pandemic on Tuberculosis Notifications, Vietnam, 2020

Appendix

Appendix Table 1. Change in monthly tuberculosis notifications between 2019 and 2020 during the COVID-19 pandemic, Vietnam*

	Vietnam			Hanoi and Ho Chi Minh City†			Da Nang and Quang Nam Provinces‡		
Mo.	2019	2020	Difference§	2019	2020	Difference§	2019	2020	Difference§
Jan	9,080	6,957	−23%	2,183	1,465	−33%	292	199	−32%
Feb	6,768	8,533	26%	1,670	1,809	8%	171	249	46%
Mar	9,259	8,252	−11%	1,879	1,653	−12%	302	228	−25%
Apr	8,698	6,217	−29%	1,854	1,359	−27%	247	222	−10%
May	9,802	8,185	−16%	1,999	1,641	−18%	301	252	−16%
Jun	8,497	8,887	5%	1,724	1,811	5%	250	261	4%
Jul	9,609	9,824	2%	2,045	1,997	−2%	265	307	16%
Aug	9,722	7,880	−19%	1,847	1,593	−14%	262	77	−71%
Sep	8,311	8,328	0%	1,731	1,689	−2%	233	198	−15%
Oct	9,663	8,618	−11%	1,956	1,861	−5%	299	256	−14%
Nov	8,577	8,051	−6%	1,862	1,787	−4%	251	246	−2%
Dec	7,694	7,266	−6%	1,766	1,584	−10%	254	220	−13%
Total	105,680	96,998	–8%	22,516	20,249	–10%	3,127	2,715	–13%

*COVID-19, coronavirus disease.
†First COVID-19 outbreak.
‡Second COVID-19 outbreak.
§Difference in number of cases reported in 2020 as a proportion of cases reported in 2019.

Appendix Table 2. Quarterly tuberculosis notifications, Vietnam, 2015–2020

	No. TB cases notified						No. MDR/RR TB cases notified					
Quarter	2015	2016	2017	2018	2019	2020	2015	2016	2017	2018	2019	2020
1	22,809	23,388	24,621	23,656	25,107	23,742	395	562	611	662	532	685
2	27,455	27,133	27,268	26,196	26,997	23,289	544	660	721	808	715	644
3	27,815	29,292	28,255	26,458	27,642	26,032	555	652	704	896	898	800
4	24,597	26,714	25,589	25,861	25,934	23,935	636	576	659	441	744	722
Total	102,676	106,527	105,733	102,171	105,680	96,998	2,130	2,450	2,695	2,807	2,889	2,851

*MDR/RR, multidrug-resistant/rifampin-resistant; TB, tuberculosis.

Appendix Table 3. Change in monthly notifications for multidrug-resistant/rifampin-resistant tuberculosis between 2019 and 2020 during the COVID-19 pandemic, Vietnam, and Hanoi and Ho Chi Minh City*

	Vietnam			Hanoi and Ho Chi Minh City †		
Month	2019	2020	Difference in 2020 as a proportion of 2019 cases	2019	2020	Difference in 2020 as a proportion of 2019 cases
Jan	222	166	−25%	107	66	−38%
Feb	106	266	151%	46	105	128%
Mar	204	253	24%	103	99	−4%
Apr	215	161	−25%	114	60	−47%
May	247	216	−13%	109	104	−5%
Jun	253	267	6%	119	131	10%
Jul	308	256	−17%	145	121	−17%
Aug	313	293	−6%	132	158	20%
Sep	277	251	−9%	114	115	1%
Oct	272	267	−2%	122	111	−9%
Nov	236	218	−8%	108	112	4%
Dec	236	237	0%	106	105	−1%
Total	2,889	2,851	–1%	1,325	1,287	–3%

*COVID-19, coronavirus disease.
†Cases in Hanoi and Ho Chi Min City were included in the total number of cases in Vietnam.

Appendix Table 4. Multidrug-resistant and rifampin-resistant tuberculosis notifications reported by the National Tuberculosis Program, Vietnam, 2015–2020

	Year					
Quarter	2015	2016	2017	2018	2019	2020
1	394	563	611	662	568	766
2	545	659	722	809	783	743
3	556	657	703	845	1,022	874
4	637	571	659	807	870	911
Total	2,132	2,450	2,695	3,123	3,243	3,294

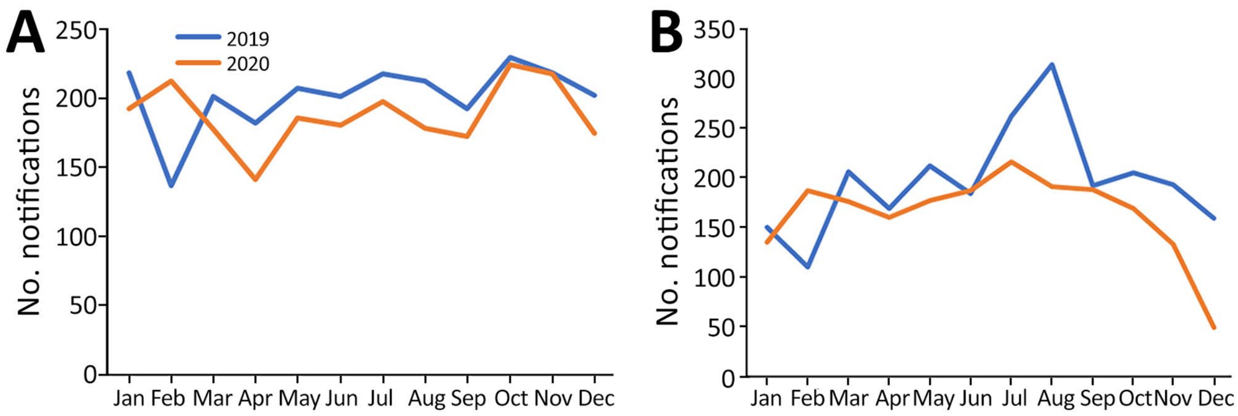

Appendix Figure. Change in monthly tuberculosis notifications between 2019 and 2020 during the coronavirus disease pandemic, Vietnam. A) Number of notifications in Can Tho. B) Number of notifications in Nghe An.
